# The New Spirit of Reciprocity

**Published:** 1921-05-07

**Authors:** 


### THE MATRONS' AND SISTERS' DEPARTMENT.

### THE NEW SPIRIT OF RECIPROCITY.

The policy of the open door which prevailed at the Conference of the General Nursing Council last week was amply justified in the result. No secrets were disclosed. Many weighty problems were discussed. Excellent organisation ensured liberty of opinion. A flood of light was thrown on the subjects discussed by the able introductory papers. And best of all the proceedings began, continued, and ended in harmony. Quite plainly the General Nursing Council is beginning to be a power.

Miss Lloyd Still spoke eloquently on the need to replace parochialism in the training-schools by nationalism, in other words to regard nursing as one great national service. Already there are numerous signs that matrons are ready to subordinate their individual opinions and methods in order to bring about reciprocity with other training-schools. The old isolated and immovable rules planned in the interest of the institution alone are giving place to considerations of a far wider reach. The training of nurses begins to adapt itself to the interests of the nurses themselves, to the interests of other institutions, to the interests of the patient generally. It is an enormous step towards the building up of a homogeneous nursing service adapted in all particulars for public needs. A system conceived in this spirit and carried out with loyalty should banish the partially trained nurse from our midst within ten years.

The first achievement of the Conference was to dispel, and this with great ease and speed, various misapprehensions which have gathered round the Draft Syllabus of the Education Committee. The syllabus was perhaps too tersely expressed for many readers. But objectors will find all criticisms fairly met in the explanations proffered by Miss Lloyd Still, and we trust that when the Draft Syllabus is no longer merely a draft, its scope may be enlarged so as to supply a commentary on the contents which will forestall future criticism.

Among the most admirable papers we would specify those of Miss Sparshott on Reciprocal Training from a General Hospital point of view, Miss Dowbiggin on the broad aspects of Poor-Law infirmary training, Miss Seymour Yapp, on linking up the Poor-Law infirmary training-schools, and Miss Villiers, on the training of fever nurses.

Miss Dowbiggin strongly emphasised the fact that the new nursing syllabus aims at abolishing the old unjustified distinction between training in a voluntary and in a Poor-Law institution. It is intended to weld together the various training-schools and give them new orientation. It is a big step in a national scheme of unification. This same note animated also the finely-sketched-out scheme for reciprocity outlined by Miss Yapp. In her indication of the lines on which the Poor-Law infirmaries could supplement each other in the nurse's training she displayed absolute freedom from the old aloof spirit of the matron of a first-class institution confronted with the difficulties of her less fortunate colleagues. She deprecates excluding even the minor Poor-Law training-schools which lack a resident medical officer from the national scheme, dreading the effects of so doing on the patients. These minor infirmaries are to take their place as "excellent first-year schools"—a courageous and fully realisable ideal. Miss Villiers neatly and lucidly summed up the advantages and disadvantages, from different points of view, in the two schemes for fever training she submitted to the conference, the one prescribing two years of fever before the general training, the other one year's fever course taken after general training.

It is sufficient to say of Miss Sparshott's proposals that if put in practice throughout the length and breadth of the land they would bring new light and vigour into small and large hospitals alike, extinguish the boredom and staleness which haunts the wards in so many institutions, close all the numerous blind alleys in the profession, and open up an inviting prospect for every probationer. It would have been a calamity had these excellent papers, to name no others, been excluded from publication by the coyness of the General Nursing Council.

